# Combined Penetrating Keratoplasty Followed by Pars Plana Vitrectomy for Firecracker Induced Bilateral Corneal and Intraocular Foreign Body: A Case Report

**DOI:** 10.31729/jnma.4932

**Published:** 2020-06

**Authors:** Lily Rajbanshi, Archana Kumari, Sanjay Singh

**Affiliations:** 1 Department of Ophthalmology, Biratnagar Eye Hospital, Rani Mills Area, Biratnagar, Nepal

**Keywords:** *eye foreign body*, *eye injuries*, *keratoplasty, penetrating*, *vitrectomy*

## Abstract

Firecracker induced open globe injury is a big challenge for ophthalmic surgeons. Its association with the intraocular foreign body makes the diagnosis and treatment even more difficult resulting in poor anatomical and visual outcomes. We report a case of a 35-year-old male who presented with bilateral, multiple corneal and intraocular foreign body due to firecracker explosion. His vision was limited to hand movement in both eyes. Combined penetrating keratoplasty and cataract surgery were done in both eyes followed by pars plana vitrectomy for intraocular foreign body removal. The final best-corrected visual acuity of the patient stood to be 6/6 and 6/9 in the right and left eye respectively. The encouraging result in our case prompts ophthalmologists for a timely stepwise multidisciplinary approach in all open globe injuries with intraocular foreign body cases having poor initial acuity.

## INTRODUCTION

Firecracker injury is a common cause of ocular trauma resulting in closed or open globe injury (OGI). Intraocular foreign body (IOFB) and globe rupture may occur with the inevitably poor visual and anatomical outcomes.^1^ Globe rupture and IOFB are known poor prognostic factors for any kind of OGI,^2^ but IOFB has differential outcomes depending on its location, nature, size, and adjacent tissue damage. However, the involvement of the posterior segment and need of multiple surgeries are definite markers of poor outcomes in case of OGI.^3^ We report a case of bilateral corneal and IOFB due to firecracker injury managed by penetrating keratoplasty (PK) and pars plana vitrectomy (PPV) in separate sittings with good outcomes in both eyes.

## CASE REPORT

A 35-year old, male visited our hospital on 11 November 2017 with a history of injury with a firecracker 4 months back. At the presentation, he had pain, redness, and diminution of vision in both eyes. His best-corrected

visual acuity (BCVA) in either eye was just hand movement on presentation. On examination, he had multiple foreign bodies (gun powder) in the conjunctiva and deep into cornea which was removed in several sittings. The patient had a history of several similar attempts in the past by his local eye surgeon. He had no known systemic illness. It was a case of open globe injury zone 1 with intraocular foreign bodies in cornea, conjunctiva, anterior chamber, lens, and vitreous cavity.

The patient had hazy cornea with peripheral vascularization and many embedded intrastromal corneal foreign bodies.

The patient was taken up for penetrating keratoplasty (PK), extracapsular cataract extraction and posterior chamber intraocular lens (PCIOL) implantation in both eyes (BE) sequentially; one-week post presentation in the left eye (LE) and 2 months later in the right eye (RE). The patient improved remarkably in spite of an episode of acute graft rejection of the left eye which was treated by an intravenous bolus of methylprednisolone, 500 mg for 3 consecutive days, and intensive topical steroids.

**Figure 1:**
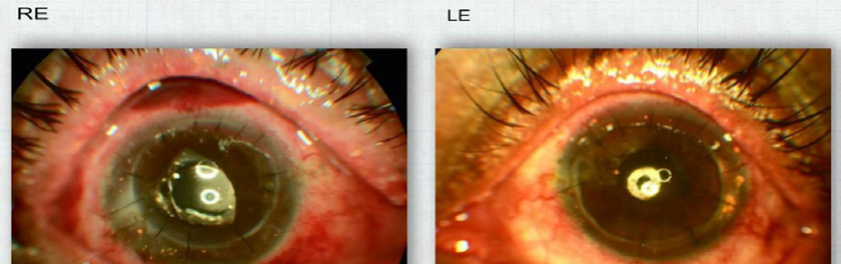
Anterior segment photograph of the right eye (RE) and left eye (LE) post penetrating keratoplasty showing clear graft with an intracorneal foreign body in host cornea.

On follow-up of four months and six months post PK, the left and right eye had BCVA of 6/36 and CF 1 m respectively (Figure 1).

**Figure 2:**
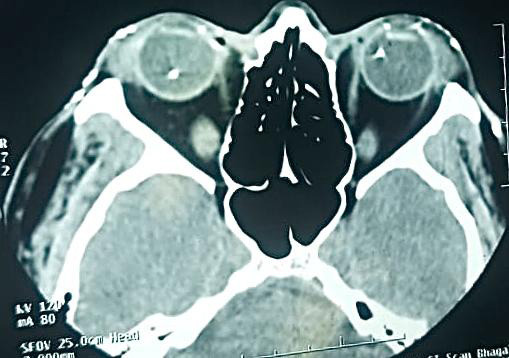
CT scan of the orbit showed multiple metallic densities in and around both orbits and bilateral maxillofacial soft tissue.

BE had clear grafts. However BE had vitritis with poor fundus view, more in LE as compared to RE. Ultrasound B scan confirmed the presence of multiple highly reflective structures in vitreous suggestive of intraocular foreign body (IOFB) along with heterogeneous vitreous echoes of moderate-intensity as seen in vitritis. CT scan of the orbit showed multiple metallic densities in and around both orbits and bilateral maxillofacial soft tissue (Figure 2).

**Figure 3:**
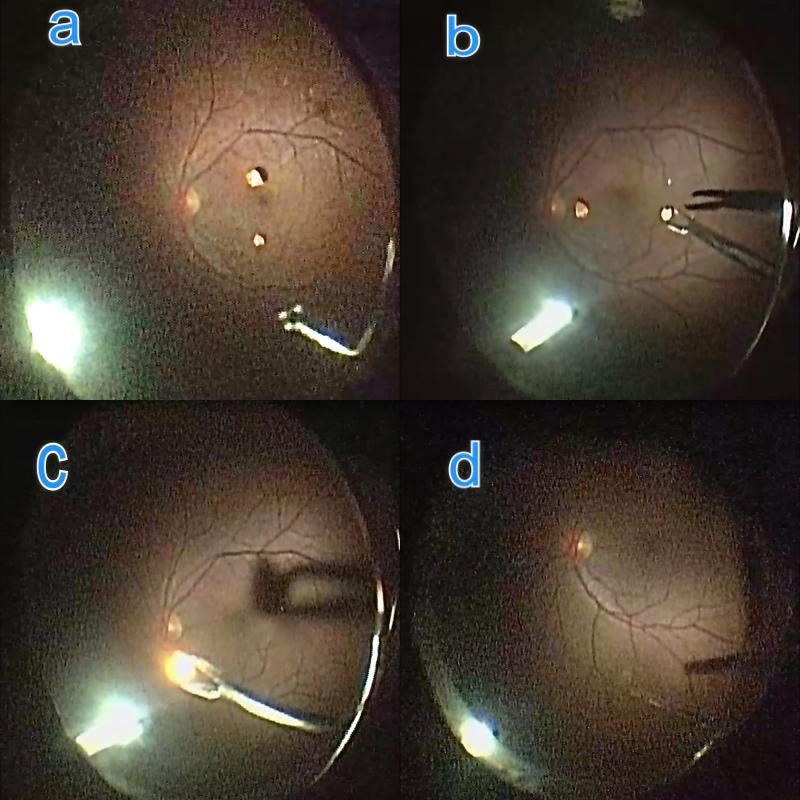
Intraoperative pars plana vitrectomy steps showing a. Intraocular foreign body (IOFB) over the retina, b. Eckardt forceps attempting to hold IOFB with caution, c. IOFB held and being removed, d. Post-IOFB removal.

On referral to vitreo retina department, we decided to take the patient for 23 gauge pars plana vitrectomy (PPV) LE in view of severe posterior segment reaction in response to IOFB. Intraoperatively brownish gunpowder clumps were seen in anterior vitreous. An attempt was made to remove it with a vitreous cutter but it fell on the retina. After inducing posterior vitreous detachment and completing a PPV attempt was made to remove IOFB with intraocular forceps taking utter care of underlying retina. IOFB dispersed into a powdery form on the pressure by the intraocular forceps and was easily removed by passive suction by flute needle. Surgery was uneventful and the eye was closed under saline. Similarly, RE underwent PPV (Figure 3).

**Figure 4:**
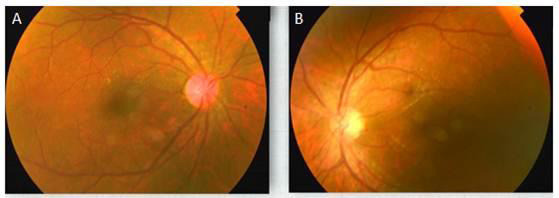
Fundus photographs of both eyes 9 months post pars plana surgery.

In this case, two solid IOFB were present which could not be removed from 23 G sclerotomy. One of the sclerotomies had to be enlarged for IOFB removal. On follow up grafts were clear and the retina was attached in BE, with no haze in vitreous cavity (Figure 4).

BCVA improved gradually over visits and his BCVA 9 months post-op in RE was 6/6 while in LE it was 6/9.

## DISCUSSION

Firecracker injury may result in posterior segment involvement in both open and closed globe injuries. However, OGI has a higher propensity of posterior segment involvement and poorer prognosis. OGI in firecracker injury may involve different zones with the incidence being 64% for zone 1, 25% for zone 2 and 11% for zone 3 with either of them resulting in posterior segment involvement.^4^ IOFB following firecracker injury may occur in as high as 12% cases and their impact on eye depend on its mechanical effect, accompanying infection and the specific reaction it triggers.^4^ Usually, high speed metallic foreign bodies are sterile and may stay innocuously in the eye until they cause a specific reaction (example, iron causing siderosis and copper causing chalcosis). It needs to be removed only if it is organic or in cases where infection or specific reaction is suspected or occurs.

One of the challenges in IOFB removal stands to be poor visibility when the cornea is involved either due to OGI repair, corneal FB, blood staining, or secondary decompensation. VR surgeons have been using PK with or without temporary keratoprosthesis in cases like vitreous hemorrhage, retinal detachment, and IOFB for good visibility during PPV. Whenever PPV is done in post PK eyes the graft clarity depends on factors like duration of surgery, post-op intraocular pressure (IOP) rise, post-op inflammation and use of tamponading agent.^5^ Silicon oil has been implicated in corneal graft endothelial damage and loss of clarity in the combined procedure of PK with PPV.^5^

In our case, the patient presented with a hazy cornea and IOFB in the vitreous cavity with minimal reaction at the onset. PK resulted in improved BCVA which was neither similar in both eyes nor persistent. Posterior segment inflammation with IOFB then prompted PPV which resulted in the bilateral improvement of VA.

The reported outcome of patients who undergo PPV in post PK eyes or combined procedure is not very good owing to the severity of primary injury-causing extensive damage, high rate of graft failure, and failure of posterior segment surgery. Graft failure in these cases are reported to be 35% by Roters et al, 51% by Watson et al. and as high as 62.7% by Lee, et al. Posterior segment surgical failure is not very high especially if there is no retinal detachment and PVR.^5^,^6^,^7^ Our reported case had post-PPV BCVA as 6/6 and 6/9 in the right and left eye respectively with the clear cornea and attached retina on followup while the BCVA at presentation was hand movement to both eyes. This is an exceptional improvement in the OGI case with PK followed by PPV. Earlier reports state regain of baseline acuity, ambulatory vision or useful vision as good as 20/400 postoperatively in OGI cases but IOFB cases in this study which was 5% had median acuity of 20/80.*^8^* Roters et al. in their study of 34 eyes had 14 IOFB injuries but none of the patients in their study had best final BCVA better than 20/650.^5^

This report states the significance of timely multidisciplinary ophthalmological interventions in firecracker injury with corneal as well as IOFB may yield unexpectedly good anatomical and functional visual outcomes.
